# A Botanical Product Containing Cistanche and Ginkgo Extracts Potentially Improves Chronic Fatigue Syndrome Symptoms in Adults: A Randomized, Double-Blind, and Placebo-Controlled Study

**DOI:** 10.3389/fnut.2021.658630

**Published:** 2021-11-26

**Authors:** Juntao Kan, Junrui Cheng, Chun Hu, Liang Chen, Siyu Liu, Dawna Venzon, Mary Murray, Shuguang Li, Jun Du

**Affiliations:** ^1^Nutrilite Health Institute, Shanghai, China; ^2^Plants for Human Health Institute, North Carolina State University, Kannapolis, NC, United States; ^3^Nutrilite Health Institute, Buena Park, CA, United States; ^4^School of Public Health, Fudan University, Shanghai, China

**Keywords:** chronic fatigue syndrome, cistanche, ginkgo, Chalder fatigue questionnaire, quality of life, sexual life quality, blood lactic acid

## Abstract

Dietary therapy may be beneficial in alleviating symptoms of chronic fatigue syndrome (CFS), a disorder that is characterized by extreme fatigue and other symptoms, but the cause of which remains unclear. The aim of this study was to evaluate the protective effect of a botanical product containing cistanche (*Cistanche tubulosa* [Schenk] Wight) and ginkgo (*Ginkgo biloba L*.) extracts on adults with CFS in a randomized, double-blind, placebo-controlled clinical trial. A total of 190 subjects (35–60 years old, non-obese) with CFS were randomized to receive one tablet of a low dose (120-mg ginkgo and 300-mg cistanche), a high dose (180-mg ginkgo and 450-mg cistanche) or a placebo once daily for 60 days. Blood samples and responses on the Chalder fatigue scale (CFQ 11), the World Health Organization's quality of life questionnaire (WHOQOL), and the sexual life quality questionnaire (SLQQ) were collected at baseline and post-intervention. CFS symptoms of impaired memory or concentration, physical fatigue, unrefreshing sleep, and post-exertional malaise were significantly improved (*p* < 0.001) in both of the treatment groups. The botanical intervention significantly decreased physical and mental fatigue scores of CFQ 11 and improved WHOQOL and SLQQ scores of the subjects (*p* < 0.01). Levels of blood ammonia and lactic acid in the treatment groups were significantly lower than those of the placebo group (low-dose: *p* < 0.05; high-dose: *p* < 0.01). In addition, the change in lactic acid concentration was negatively associated with the severity of CFS symptoms (*p* = 0.0108) and was correlated with the change in total physical fatigue score of the CFQ (*p* = 0.0302). Considering the trivial effect size, the results may lack clinical significance. In conclusion, this botanical product showed promising effects in ameliorating the symptoms of CFS. Clinical trials with improved assessment tools, an expanded sample size, and an extended follow-up period are warranted to further validate the findings.

**Clinical Trial Registration:** https://clinicaltrials.gov/, identifier: NCT02807649.

## Introduction

Chronic fatigue syndrome (CFS) is the common name for a group of significantly debilitating medical conditions characterized by persistent fatigue and other specific symptoms that last for a minimum of 6 months in adults. The condition occurs predominantly in women (1). CFS symptoms include post-exertional malaise, unrefreshing sleep, impaired memory or concentration, muscle pain, polyarthralgia, sore throat, tender lymph nodes, and new headaches (2). The global prevalence of CFS is increasing, at present estimated to be between 0.4 and 2.5% (3). The cause of CFS is still unclear, but the condition appears to result from multiple factors. Generally, the onset of CFS is associated with psychological stress, endocrine and immune disorders, genetic factors, and viral infections (1). From a mechanistic perspective, CFS may be the result of inflammation, elevated oxidative stress, and impaired redox status, factors that consequently lead to acquired mitochondrial dysfunction, abnormalities in the hypothalamic-pituitary-adrenal axis, and abnormal adrenergic metabolism (4). In addition, CFS patients may experience abnormally high lactate levels and slowed acid clearance in muscle due to their impaired mitochondrial function (5); therefore, a high blood lactic acid level is often seen in subjects with CFS (4). Currently, there is no standard treatment for CFS. Commonly used treatments include medications that enhance the immune system, nutrition–balanced therapy, cognitive behavioral therapy, local physiotherapy, and graded exercise therapy (6). The main purposes of the treatments are to relieve CFS symptoms, improve psychological well-being, and restore social behavioral functions.

A high prevalence of nutritional supplement usage has been reported among CFS patients, and half of the patients in one study benefitted from nutritional intervention (7). Therefore, dietary therapy such as diet modification and dietary supplementation may be beneficial in alleviating symptoms and reducing fatigue in CFS patients (8). Ginkgo is an extract from the leaves of *Ginkgo biloba* L. Ginkgo is a powerful antioxidant that can improve cerebral perfusion and associated memory and cognitive deficits (8). Ginkgo supplementation was found to be effective in clearing free radicals in mice during endurance exercise by enhancing superoxide dismutase activity, which delayed fatigue (9). Intriguingly, ginkgo extract supplementation at 120 mg/d also significantly alleviated psychological and physiological distress and fatigue among subjects at a high risk of developing mental health disorders (10).

Cistanche (*Cistanche tubulosa* [Schenk] Wight) is a popular dietary and traditional ingredient (TCM) in Chinese traditional medicine. In an *in vivo* study, phenylethanoid-rich extracts of Cistanche at 0.25, 0.50, and 1.00 g/kg bw significantly decreased serum lactic acid levels and delayed the onset of fatigue (11). Based on traditional Chinese medicine (TCM) theory, a healthy status stems from the perfect balance of a set of opposing forces, namely *Yin* and *Yang* (12). *Yang* governs vigor, meaning that Yang is the driving force of biological activities in the human body (13), whereas *Yin* governs rest, facilitating relaxation and tissue repair (12). Since *Yang* plays a vital role in maintaining a normal metabolic rate and energizing the body, deficiencies in *Yang* may lead to fatigue syndromes in humans (13). As a *Yang*-invigorating tonic herb of TCM (i.e., a herb that reinforces *Yang*), cistanche has been demonstrated to be beneficial in the treatment of CFS in subjects with *Yang* deficiency through enhancing mitochondrial function (12). TCM considers the balance of *Yin* and *Yang* as a critical factor in maintaining a harmonious status between body, mind, and spirit, and the homeostasis of *Yin*- and *Yang*-balance acts as a foundation for overall wellness (12).

A botanical combination of cistanche and ginkgo extract has been developed and shown to improve memory in healthy subjects (Patent ID US9737582B2, US20150320818A1). However, whether such a product exhibits potential health benefits regarding CFS symptoms remains unknown. Therefore, we examined the effect of a cistanche/ginkgo product on the CFS symptoms in a randomized, double-blind, placebo-controlled trial.

## Methods

### Trial Design

**Figure 1:**
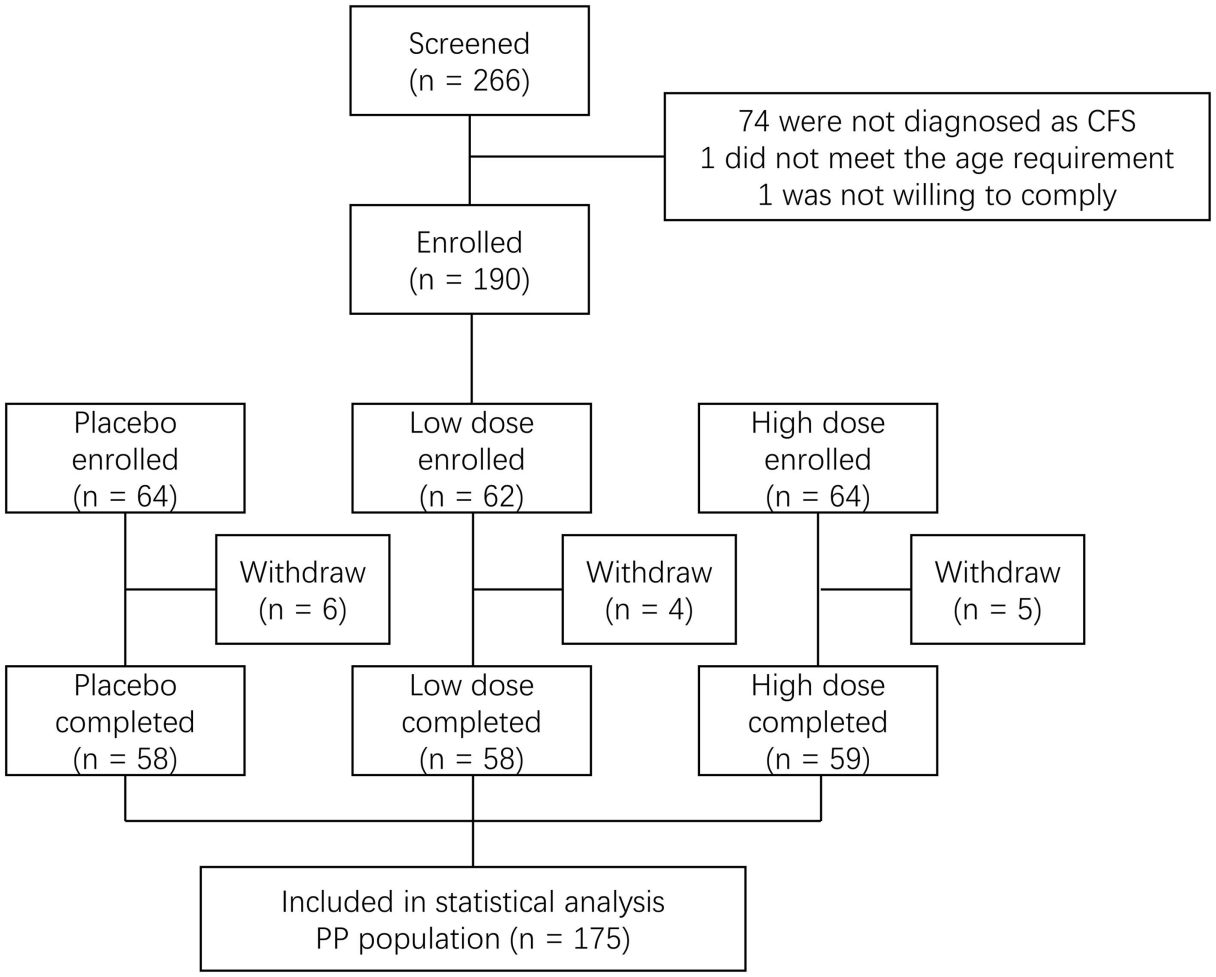
Flowchart of the clinical trial.

The clinical trial was conducted in Community Hospital of Baoshan District (Shanghai, China) from May to December 2015. The subjects were identified from a hospital-owned database. A total of 190 subjects were initially enrolled in the study at baseline and were classified by age and gender according to a population-based study in China (14) as (1) males aged 35–50 years old and females aged 35–40 years old; (2) males aged 51–60 years old and females aged 41–60 years old. The subjects were randomly assigned to three groups (group 1: placebo; group 2: a low dose of the product; group 3: a high dose of the product). The subjects consumed their designated products once daily for 60 consecutive days. During the study, 15 subjects withdrew, leaving 175 subjects in the per protocol (PP) analysis (Figure 1). The total dropout rate was 7.9%. Blood samples and questionnaires of Chalder fatigue, quality of life (QOL), and sexual life quality (SLQ) were collected at baseline and post-intervention (day 60). The study was conducted according to the guidelines laid down in the 1964 Declaration of Helsinki and its later amendments. Informed consent was obtained from all subjects. This study was approved by the Institutional Review Board (IRB) of Shanghai Nutrition Society and registered at http://clinicaltrials.gov (NCT02807649).

### Randomization

Prior to randomization, all inclusion and exclusion criteria were verified to ensure that the potential volunteers qualified for study enrollment. Randomized sequences were generated by using a sequence generator (https://www.random.org/sequences/). The opaque, sequentially numbered randomization envelopes remained sealed until a subject was determined to be eligible for enrollment. To ensure balanced allocation for subjects between different genders in different age groups, the randomization number for the subject was determined as a three-digit random number.

The information requested on the randomization sheet (e.g., subject initials, birth date, and informed consent date) was recorded. The randomization sheets were retained at the study site. The researchers who assigned subjects were not given access to the study site before assignment to ensure that they did not have access to the randomization/allocation documents. The product assignment and subject code (i.e., random number) appearing on the randomization sheet were recorded on the appropriate case report form. For the personal information to remain confidential, each subject was identified only by initials, birth date, and random number.

### Intervention

According to a previous report, a single-dose supplementation of ginkgo extract at 120 mg significantly improved cognition and memory performance (15). Cistanche extract supplementation for 48 weeks at 300 mg/d was effective in improving cognitive function in subjects with moderate Alzheimer's disease (16). Based on these data, we hypothesized that a combination of 300-mg cistanche and 120-mg ginkgo may alleviate subjects' mental fatigue, and a higher dosage (a 50% increase) may further improve the beneficial effects. Therefore, the test products were tablets containing a water extract of *Cistanche tubulosa* root (300 mg per dose for the low-dose group and 450 mg per dose for the high-dose group; minimum 28% echinacoside) and water extract of *Ginkgo biloba* leaf (120 mg per dose for the low-dose group and 180 mg per dose for the high-dose group; 24% total flavonol glycoside). The *Ginkgo biloba* leaf extract was obtained from Ningbo Green-Health Pharmaceutical Co., Ltd. (Ningbo, Zhejiang, China), and *Cistanche tubulosa* root extract was provided by Sinphar Tianli Pharmaceutical Co., Ltd. (Hangzhou, Zhejiang, China). The excipients of the tablets include microcrystalline cellulose, dextrose, maltodextrin, corn starch, croscarmellose sodium, silicon dioxide, magnesium stearate, and coating agents.

Subjects were randomized to receive three tablets of the low dose, high dose, or placebo (tablets with excipients only) once daily for 60 days. The tablets given to the subjects had the same taste and appearance. All the test products and the placebo were manufactured in a Good Manufacturing Practice pilot plant and manufactured (Nutrilite) under quality assurance to ensure compliance with the regulatory requirements for microbial, heavy metal, and pesticide levels.

### Inclusion/Exclusion Criteria

The inclusion criteria were as follows: both male and female volunteers (between 35 and 60 years of age) who were diagnosed as having CFS by a study physician (Additional details on the assessment of CFS are provided in the following section). Subjects with BMI (body mass index) ≥ 28 kg/m^2^ were excluded due to the correlation between obesity and fatigue (17), with obesity being defined as BMI ≥ 28 kg/m^2^ (18, 19). Subjects were excluded if they had flulike or other symptoms of viral infection within 3 months prior to the first visit to the clinical site, or if they had a history of or had been diagnosed with any of the following diseases that might affect the study results: gastrointestinal disorders, skeletal muscle dysfunction, hepatopathy, nephropathy, endocrine disease, blood disorders, respiratory and cardiovascular diseases. Subjects were also excluded if they were currently taking medicine for cardiovascular or metabolic diseases; if they were current smokers or current or previous alcohol abusers; if they were pregnant or lactating; if they were currently having or had any medical or nutritional therapies, including taking protein supplements or nutrients that promoted exercise capacity within 3 months before screening; if they had lost or gained weight over 5 kg within 3 months before screening; if they had been hospitalized within 3 months before screening; if they had participated in similar clinical trials within 6 months before screening; or if they were unwilling to comply with the study procedures.

### CFS Assessment

The CFS was assessed at enrollment in accordance with the diagnostic criteria published by the U.S. Centers for Disease Control (CDC) (2). CFS was diagnosed when (1) the continuous or recurrent attacks of unexplained severe fatigue lasted for more than 6 months and could not be alleviated after sufficient rest, resulting in substantial reduction in previous levels of occupational, educational, social, or personal activities; (2) four or more of the following symptoms were concurrently present for over 6 months: impaired memory or concentration; sore throat; tender cervical or axillary lymph nodes; muscle pain; multiple joint pain; new headaches; unrefreshing sleep; and post-exertional malaise. After the intervention, each of the above symptoms was assessed again for evaluation of the product efficacy. The overall effective evaluation was based on the sum of remission cases (the majority of symptoms were relieved) and relived cases (some symptoms were relieved).

### Chalder Fatigue Questionnaire

The Chalder fatigue questionnaire has been widely used to measure the extent and severity of physical and mental fatigue in clinical and epidemiological studies. The original Chalder fatigue scale consisted of 14 items designed to measure fatigue severity over the past 3 months (20). In the following studies, three items were dropped from the 14-item version, resulting in a revised 11-item version (21). For the 11 items, there are two subscales to evaluate two types of fatigue: physical and mental. The scores are rated on a four-point Likert scale (0 = not at all; 1 = the same as usual; 2 = more than usual; 3 = much more than usual). A higher score indicates a greater level of fatigue.

### QOL Questionnaire

The QOL was assessed using a modified World Health Organization (WHO) QOL questionnaire (22, 23). The questionnaire comprised 26 items, providing an integrated measurement of an individual's QOL based on the following factors: physical health (PHYS), psychological health (PSYCH), social relationships (SOCIL), living environment (ENVIR), and subjects' self-evaluation of their QOL and health status (Q1 and Q2). Each item in the questionnaire provided a score ranging from 1 to 5. The scores of Q3, Q4, and Q26 were taken inversely (the new score was the difference between 6 and the original score) since the higher original scores were associated with lower QOL. The formula for the QOL score calculation was listed as follows: PHYS = 4 × [(6–Q3) + (6–Q4)+Q10+Q15+Q16+Q17+Q18]/7; PSYCH = 4 × [Q5+Q6+Q7+Q11+Q19+(6–Q26)]/6; SOCIL = 4 × (Q20+Q21+Q22)/3; ENVIR = 4 × (Q8+Q9+Q12+Q13+Q14+Q23+Q24+Q25)/8; QOL score = (Q1+Q2+PHYS+PSYCH+SOCIL+ENVIR-4) × (100/16).

### SLQ Questionnaire

The SLQ questionnaire was previously developed to evaluate the quality of sexual life of the subjects (24). The original 10 item scores (ranging from −4 to +4 in value) were converted to a 0 to 8 scale score by adding 4 to each recorded response. The 0 to 8 scale score was converted to a standardized score by dividing that score by 8 and multiplying the result by 100. The SLQ scale score for a respondent was computed as the mean of the 10 standardized item scores.

### Biochemical Analysis

Blood ammonia, glucose, free fatty acid, creatine kinase, C-reactive protein, lactic acid, estradiol (only for females), and testosterone (only for males) were determined using commercially available kits according to the manufacturer's instructions (Jiancheng, Nanjing, China). The levels of alanine aminotransferase (ALT), aspartate transaminase (AST), gamma-glutamyl transferase (GGT), and blood urea nitrogen (BUN) were determined by using commercially available colorimetric detection kits (Jiancheng, Nanjing, China).

### Sample Size Calculation

The sample size was chosen based on a significance level of 0.016 (0.05 after adjustment for three multiple comparisons), 80% power, and expected proportions of respondents of 0.5 and 0.8 for the placebo and intervention groups, respectively. Sixty-three participants for each group would yield 53 completed participants assuming a 15% dropout rate. A total of 190 subjects were enrolled to ensure at least 159 subjects that completed the study.

### Statistical Analysis

Statistical analyses were performed using SAS 9.4 (SAS Institute Inc., Cary, NC, USA). All statistical tests of the hypotheses were two-sided and were performed at the 0.05 significance level. Mean and standard deviations were summarized for normally distributed continuous variables; medians were provided for non-normal variables, and frequencies and percentages were provided for categorical variables. Evaluations of product effect were performed using chi-squared tests or analysis of covariance (ANCOVA) followed by pairwise group comparisons for the variables with significant group differences.

## Results

### Characteristics of Subjects

Summary statistics of baseline subject characteristics are shown in Table 1. No significant differences were observed at baseline among groups in demographic data, including gender, age, body weight, height, body mass index, body temperature, and blood pressure. Subjects' compliance was monitored and verified by the number of unused products returned to the study site. All 175 subjects who completed the study consumed all assigned products.

**Table 1:** Baseline characteristics of subjects who completed the study.

**Baseline characteristics**	**Placebo (*n* = 58)**	**Low dose (*n* = 58)**	**High dose (*n* = 59)**	***p*-value**
Female (*n*)	29 (50.0%)	31 (53.4%)	31 (52.5%)	0.928
Gender and age strata				0.894
Female age 35–40 (*n*)	6 (10.3%)	4 (6.9%)	5 (8.5%)	N/A
Female age 41–60 (*n*)	23 (39.7%)	27 (46.6%)	26 (44.1%)	N/A
Male age 35–50 (*n*)	6 (10.3%)	9 (15.5%)	6 (10.2%)	N/A
Male age 51–60 (*n*)	23 (39.7%)	18 (31.0%)	22 (37.2%)	N/A
Age (years)	50.7 ± 7.6	51.5 ± 7.5	50.5 ± 7.0	0.737
Weight (kg)	66.0 ± 11.0	64.6 ± 9.0	65.5 ± 10.8	0.741
Height (cm)	166.4 ± 7.4	164.9 ± 7.2	165.4 ± 8.2	0.553
BMI (kg/m^2^)	23.8 ± 2.8	23.7 ± 2.6	23.9 ± 2.9	0.963
Body temperature (°C)	36.8 ± 0.2	36.8 ± 0.2	36.8 ± 0.3	0.715
Systolic pressure (mmHg)	123.8 ± 10.8	123.1 ± 14.4	121.8 ± 11.8	0.687
Diastolic pressure (mmHg)	76.5 ± 6.1	76.1 ± 8.2	74.0 ± 9.2	0.200

*Data are number of patients (n), frequency (%) or mean ± standard deviation (SD). Group differences were evaluated using a chi-square test or one-way analysis of variance (ANOVA). N/A, not applicable*.

### CFS Assessment

The frequency and percentage of subjects with remission or relieved CFS as well as the total numbers of effective cases after intervention are shown in Table 2. Nine (15.5%) subjects in the low-dose group had significantly improved CFS; 11 (18.6%) subjects improved in the high-dose group compared to 0% in the placebo group. Partial relief of symptoms was observed in 33 subjects or 56.9%, 37 subjects or 62.7%, and 16 subjects or 27.6% in the low-dose, high-dose, and placebo groups, respectively. These findings led to an overall effectiveness of 72.4% in the low-dose group and 81.4% in the high-dose group (*P* < 0.001) compared to 27.6% in the placebo group. The effectiveness of the product was not different between genders. Among all eight individual symptoms, impaired memory or concentration, muscle pain, unrefreshing sleep, and post-exertional malaise were significantly (*P* < 0.001) relieved in the subjects of the low-dose and high-dose groups compared to those in the placebo group after product intervention (Supplementary Table 1).

**Table 2:** Summary of the change of symptoms of chronic fatigue syndrome (CFS).

	**Placebo (*n* = 58)**	**Low dose (*n* = 58)**	**High dose (*n* = 59)**	**Product effect *p*-value**
Remission (*n*)	0 (0.0%)	9 (15.5%)^**^	11 (18.6%)^**^	**0.0032**
Relief (*n*)	16 (27.6%)	33 (56.9%^)**^	37 (62.7%)^***^	**0.0004**
Treatment responders (*n*)	16 (27.6%)	42 (72.4%)^***^	48 (81.4%)^***^	** <0.0001**

*Data are number of patients (n) or frequency (%). Product effect was evaluated by chi-square test. Post-hoc test with Bonferroni adjustment was applied to variables with significant group difference for further pair-wise group comparison*.

** *P < 0.01 and*

*** *P < 0.001 compared with placebo group. Numbers in bold indicate statistical significance*.

### Chalder Fatigue Questionnaire

The score of each question in the 11-item Chalder fatigue questionnaire and the total physical and mental fatigue scores are summarized in Table 3. After 60 days of product intervention, both low-dose and high-dose groups had significantly lower fatigue scores compared to the placebo group for all physical and mental fatigue questions (*P* < 0.001). Significant differences were also observed for the low-dose group vs. the high-dose group in total physical fatigue score (*P* < 0.01) and Q3-sleepy or drowsy feeling score (*P* < 0.01), suggesting a superior effectiveness of the high-dose treatment. The changes in the scores were comparable between males and females.

**Table 3:** Changes in the scores of Chalder fatigue questionnaire.

	**Baseline**			**Day 60**			**Product effect *p*-value**
	**Placebo (*n* = 58)**	**Low dose (*n* = 58)**	**High dose (*n* = 59)**	**Placebo (*n* = 58)**	**Low dose (*n* = 58)**	**High dose (*n* = 59)**	
**Physical fatigue (total score):**	9.93 ± 2.25	9.78 ± 1.86	9.92 ± 1.98	9.64 ± 1.61	6.59 ± 1.96^***^	5.61 ± 2.39^***^^, *^##^*^	** <0.0001**
1. Do you have problems with tiredness?	1.50 ± 0.54	1.53 ± 0.57	1.46 ± 0.60	1.45 ± 0.50	0.83 ± 0.63^***^	0.66 ± 0.58^***^	** <0.0001**
2. Do you need to rest more?	1.45 ± 0.54	1.47 ± 0.57	1.51 ± 0.54	1.36 ± 0.55	0.90 ± 0.52^***^	0.75 ± 0.54^***^	** <0.0001**
3. Do you feel sleepy or drowsy?	1.43 ± 0.57	1.45 ± 0.50	1.49 ± 0.50	1.38 ± 0.64	0.95 ± 0.60^***^	0.66 ± 0.58^***^^, *^##^^##^*^	** <0.0001**
4. Do you have problems starting things?	1.19 ± 0.44	1.10 ± 0.31	1.15 ± 0.36	1.22 ± 0.50	0.95 ± 0.22^***^	0.93 ± 0.31^***^	** <0.0001**
5. Do you lack energy?	1.41 ± 0.56	1.34 ± 0.48	1.37 ± 0.49	1.36 ± 0.64	0.91 ± 0.47^***^	0.71 ± 0.53^***^	** <0.0001**
6. Do you have less strength in your muscles?	1.57 ± 0.53	1.48 ± 0.57	1.53 ± 0.63	1.52 ± 0.54	1.19 ± 0.48^***^	1.12 ± 0.49^***^	** <0.0001**
7. Do you feel weak?	1.38 ± 0.59	1.40 ± 0.49	1.41 ± 0.50	1.34 ± 0.55	0.86 ± 0.44^***^	0.78 ± 0.46^***^	** <0.0001**
**Mental fatigue (total score):**	5.52 ± 1.33	5.38 ± 1.12	5.41 ± 1.08	5.29 ± 1.24	3.59 ± 1.04^***^	3.37 ± 1.07^***^	** <0.0001**
8. Do you have difficulties concentrating?	1.43 ± 0.53	1.40 ± 0.49	1.42 ± 0.50	1.33 ± 0.51	0.74 ± 0.52^***^	0.71 ± 0.53^***^	** <0.0001**
9. Do you make slips of the tongue when speaking?	1.16 ± 0.41	1.14 ± 0.40	1.17 ± 0.38	1.19 ± 0.51	0.91 ± 0.34^***^	0.88 ± 0.38^***^	** <0.0001**
10. Do you find it more difficult to find the correct word?	1.24 ± 0.47	1.22 ± 0.42	1.20 ± 0.41	1.21 ± 0.52	0.95 ± 0.22^***^	0.93 ± 0.25^***^	** <0.0001**
11. How is your memory?	1.69 ± 0.54	1.62 ± 0.52	1.61 ± 0.49	1.57 ± 0.62	0.98 ± 0.58^***^	0.85 ± 0.61^***^	** <0.0001**

*Data are mean ± SD. Each question was rated by scores. Higher scores represent higher possibility. For item Q11, a higher score represents better memory. Product effect was evaluated by analysis of covariance (ANCOVA). Post-hoc test with Bonferroni adjustment was applied to variables with significant group difference for further pair-wise group comparison at day 60*.

*** *P < 0.001 compared with placebo group*,

## *P < 0.01 compared with low dose group. Numbers in bold indicate statistical significance*.

### QOL and SLQ Questionnaires

The individual and area (PHYS, PSYCH, SOCIL, and ENVIR) scores of the QOL questionnaire are shown in Table 4. After 60 days of product intervention, a significant group difference was observed in Q1-overall rating of quality of life and Q2-overall satisfaction of health; in Q3, 10, 16, and 17 under the PHYS area as well as the area score; in Q7 and 26 under the PSYCH area, and Q21 under the SOCIL area.

**Table 4:** Changes in the scores of quality of life (QOL) questionnaire.

	**Baseline**			**Day 60**			**Product effect *p*-value**
	**Placebo (*n* = 58)**	**Low dose (*n* = 58)**	**High dose (*n* = 59)**	**Placebo (*n* = 58)**	**Low dose (*n* = 58)**	**High dose (*n* = 59)**	
							
1. How would you rate your quality of life?	3.4 ± 0.6	3.4 ± 0.6	3.4 ± 0.6	3.4 ± 0.5	3.7 ± 0.5^*^	3.8 ± 0.5^***^	**0.0021**
2. How satisfied are you with your health?	3.3 ± 0.7	3.2 ± 0.7	3.3 ± 0.6	3.2 ± 0.6	3.6 ± 0.6^***^	3.7 ± 0.6^***^	** <0.0001**
Physics (PHYS area score):	48.8 ± 8.4	49.2 ± 7.4	48.1 ± 8.4	49.1 ± 7.3	52.6 ± 7.5^*^	53.8 ± 8.6^***^	**0.0024**
3. To what extent do you feel that physical pain prevents you from doing what you need to do?	2.1 ± 0.8	2.1 ± 0.7	2.1 ± 0.9	2.2 ± 0.9	1.8 ± 0.9^*^	1.7 ± 0.8^**^	**0.0034**
4. How much do you need any medical treatment to function in your daily life?	1.7 ± 0.8	1.8 ± 0.9	1.6 ± 0.9	1.6 ± 0.9	1.6 ± 1.0	1.7 ± 1.0	0.8198
10. Do you have enough energy for everyday life?	3.2 ± 0.7	3.2 ± 0.7	3.1 ± 0.7	3.2 ± 0.7	3.5 ± 0.7^*^	3.6 ± 0.6^**^	**0.0034**
15. How well are you able to get around?	3.6 ± 0.6	3.6 ± 0.6	3.6 ± 0.7	3.6 ± 0.5	3.7 ± 0.6	3.8 ± 0.6	0.1882
16. How satisfied are you with your sleep?	3.0 ± 0.7	2.9 ± 0.9	2.9 ± 0.8	3.1 ± 0.7	3.6 ± 0.8^***^	3.8 ± 0.7^***^	** <0.0001**
17. How satisfied are you with your ability to perform your daily living activities?	3.5 ± 0.6	3.6 ± 0.7	3.5 ± 0.6	3.4 ± 0.6	3.8 ± 0.5^**^	3.8 ± 0.5^***^	**0.0007**
18. How satisfied are you with your capacity for work?	3.6 ± 0.6	3.6 ± 0.6	3.6 ± 0.6	3.6 ± 0.6	3.7 ± 0.5	3.7 ± 0.5	0.2374
Psychology (PSYCH area score):	54.0 ± 10.2	54.0 ± 10.1	54.3 ± 9.5	56.3 ± 8.8	57.3 ± 8.5	57.8 ± 8.0	0.4383
5. How much do you enjoy life?	3.3 ± 0.7	3.3 ± 0.9	3.3 ± 0.8	3.3 ± 0.6	3.5 ± 0.6	3.6 ± 0.5	0.0536
6. To what extent do you feel your life to be meaningful?	3.4 ± 0.8	3.4 ± 0.8	3.4 ± 0.7	3.6 ± 0.6	3.6 ± 0.6	3.6 ± 0.5	0.8199
7. How well are you able to concentrate?	3.0 ± 0.8	3.0 ± 0.7	3.0 ± 0.7	3.1 ± 0.7	3.4 ± 0.7^**^	3.5 ± 0.7^***^	**0.0004**
11. Are you able to accept your bodily appearance?	3.4 ± 0.6	3.4 ± 0.5	3.4 ± 0.7	3.5 ± 0.6	3.6 ± 0.6	3.6 ± 0.6	0.8489
19. How satisfied are you with yourself?	3.6 ± 0.6	3.6 ± 0.6	3.6 ± 0.6	3.6 ± 0.6	3.7 ± 0.5	3.7 ± 0.5	0.3861
26. How often do you have negative feelings, such as blue mood, despair, anxiety, depression?	2.3 ± 0.7	2.3 ± 0.7	2.3 ± 0.8	2.4 ± 0.5	2.0 ± 0.5^***^	1.9 ± 0.5^***^	** <0.0001**
Social (SOCIL area score):	59.6 ± 11.0	58.8 ± 13.1	59.7 ± 10.8	60.9 ± 10.9	64.2 ± 10.9	65.1 ± 9.1	0.0654
20. How satisfied are you with your personal relationships?	3.6 ± 0.6	3.6 ± 0.6	3.6 ± 0.6	3.6 ± 0.6	3.7 ± 0.5	3.6 ± 0.6	0.8873
21. How satisfied are you with the support you get from your friends?	3.1 ± 0.8	3.0 ± 0.7	3.0 ± 0.8	3.2 ± 0.7	3.5 ± 0.7^**^	3.6 ± 0.6^***^	**0.0004**
22. How satisfied are you with your performance at the party?	3.5 ± 0.5	3.5 ± 0.6	3.6 ± 0.6	3.6 ± 0.5	3.6 ± 0.6	3.6 ± 0.5	0.9989
Environment (ENVIR area score):	54.7 ± 11.2	54.6 ± 11.4	54.6 ± 11.3	55.5 ± 7.2	56.0 ± 8.4	56.1 ± 9.9	0.9055
8. How safe do you feel in your daily life?	3.1 ± 0.7	3.2 ± 0.7	3.2 ± 0.7	3.2 ± 0.5	3.2 ± 0.5	3.3 ± 0.7	0.7612
9. How healthy is your physical environment?	2.9 ± 0.8	2.9 ± 0.8	2.9 ± 0.7	3.1 ± 0.6	3.1 ± 0.7	3.0 ± 0.7	0.9755
12. Have you enough money to meet your needs?	3.1 ± 0.5	3.2 ± 0.9	3.1 ± 0.7	3.2 ± 0.8	3.2 ± 0.8	3.2 ± 0.7	0.9768
13. How available to you is the information that you need in your day-to-day life?	3.3 ± 0.6	3.3 ± 0.6	3.3 ± 0.6	3.3 ± 0.5	3.3 ± 0.6	3.3 ± 0.6	0.8769
14. To what extent do you have the opportunity for leisure activities?	3.2 ± 0.9	3.2 ± 0.8	3.1 ± 0.9	3.2 ± 0.8	3.2 ± 0.8	3.2 ± 0.8	0.8395
23. How satisfied are you with the conditions of your living place?	3.3 ± 0.7	3.3 ± 0.7	3.3 ± 0.7	3.3 ± 0.6	3.3 ± 0.5	3.3 ± 0.5	0.9487
24. How satisfied are you with your access to health service?	3.2 ± 0.6	3.2 ± 0.7	3.2 ± 0.7	3.2 ± 0.5	3.3 ± 0.6	3.3 ± 0.7	0.8256
25. How satisfied are you with your transport?	3.3 ± 0.5	3.3 ± 0.6	3.3 ± 0.7	3.3 ± 0.5	3.3 ± 0.6	3.3 ± 0.6	0.9248

*Data are mean ± SD. Each question was rated by scores. Higher scores represent higher possibility. The item score of Q3, Q4, and Q26 were reversed (calculated as 6 minus raw item score). Product effect was evaluated by ANCOVA. Post-hoc test with Bonferroni adjustment was applied to variables with significant group difference for further pair-wise group comparison at day 60*.

* *P < 0.05*,

** *P < 0.01, and*

*** *P < 0.001 compared with placebo group. Numbers in bold indicate statistical significance*.

The individual and scale scores of the SLQ questionnaire with stratification by gender are shown in Table 5. For male subjects, there were significant group differences in all 10 items and scale scores after the product intervention. For female subjects, significant group differences were observed in all items except ease of insertion and partner's overall pleasure of intercourse.

**Table 5:** Changes in the scores of sexual life quality (SLQ) questionnaire.

**Gender**		**Baseline**			**Day 60**			**Product effect *p*-value**
		**Placebo (*n* = 58)**	**Low dose (*n* = 58)**	**High dose (*n* = 59)**	**Placebo (*n* = 58)**	**Low dose (*n* = 58)**	**High dose (*n* = 59)**	
Male	1. Frequency of lovemaking	43.8 ± 17.2	46.3 ± 17.3	42.4 ± 14.6	46.1 ± 13.8	59.7 ± 14.0^***^	63.8 ± 11.5^***^	** <0.0001**
	2. Duration of lovemaking	42.9 ± 15.0	42.1 ± 10.5	39.3 ± 11.1	48.7 ± 11.3	63.9 ± 14.0^***^	62.1 ± 12.0^***^	** <0.0001**
	3. Ease of insertion	48.2 ± 11.1	49.5 ± 11.2	48.7 ± 12.4	52.2 ± 14.6	60.6 ± 12.4^*^	60.7 ± 10.0^**^	**0.0106**
	4. Ease of achieving orgasm	49.6 ± 14.6	50.0 ± 17.3	47.8 ± 15.6	53.0 ± 13.6	62.0 ± 13.6^*^	62.9 ± 14.2^**^	**0.0150**
	5. Ease of initiating lovemaking	52.2 ± 13.2	51.4 ± 20.0	49.6 ± 15.4	52.2 ± 11.1	64.4 ± 15.0^***^	62.9 ± 11.0^***^	** <0.0001**
	6. Pleasure of anticipation	50.0 ± 9.0	51.4 ± 17.8	52.2 ± 15.2	47.8 ± 13.8	63.0 ± 14.1^***^	68.3 ± 13.8^***^	** <0.0001**
	7. Carefree feelings during lovemaking	46.9 ± 11.1	45.8 ± 15.5	46.0 ± 10.8	53.9 ± 12.5	66.7 ± 15.9^**^	66.1 ± 13.1^**^	**0.0013**
	8. Pleasure of orgasm	42.4 ± 10.9	39.4 ± 17.6	38.8 ± 12.9	50.0 ± 14.9	61.6 ± 14.7^**^	62.1 ± 12.9^**^	**0.0018**
	9. Overall pleasure of lovemaking	43.8 ± 11.0	43.5 ± 18.1	42.0 ± 13.3	52.2 ± 13.0	63.0 ± 16.4^**^	63.4 ± 13.6^**^	**0.0065**
	10. Partner's overall pleasure of lovemaking	46.9 ± 10.0	47.2 ± 17.1	46.0 ± 11.3	52.6 ± 12.2	59.7 ± 14.8^*^	60.7 ± 12.1^*^	**0.0348**
	SLQ scale score (mean of item 1-10)	46.7 ± 8.3	46.7 ± 12.1	45.3 ± 10.3	50.9 ± 9.9	62.5 ± 12.3^***^	63.3 ± 10.4^***^	** <0.0001**
Female	1. Frequency of lovemaking	38.4 ± 19.8	36.3 ± 14.1	40.7 ± 17.7	36.5 ± 16.1	52.8 ± 15.2^***^	63.4 ± 16.0^***^	** <0.0001**
	2. Duration of lovemaking	45.1 ± 21.1	43.3 ± 12.6	46.8 ± 18.0	47.0 ± 18.8	56.0 ± 19.4^*^	61.2 ± 17.8^**^	** <0.0001**
	3. Ease of insertion	46.4 ± 18.9	44.6 ± 9.7	46.8 ± 16.1	50.5 ± 18.2	57.4 ± 16.4	58.2 ± 16.8	0.0909
	4. Ease of achieving orgasm	38.8 ± 17.8	40.0 ± 13.3	42.3 ± 13.2	42.0 ± 18.4	52.3 ± 15.1^*^	53.9 ± 15.0^*^	**0.0240**
	5. Ease of initiating lovemaking	42.0 ± 17.1	42.5 ± 13.0	44.0 ± 13.6	46.5 ± 16.7	56.0 ± 17.5	64.7 ± 19.8^***^	**0.0003**
	6. Pleasure of anticipation	49.1 ± 13.6	47.5 ± 10.1	46.4 ± 14.1	50.5 ± 16.3	52.8 ± 12.7	62.5 ± 19.2^**^	**0.0042**
	7. Carefree feelings during lovemaking	47.8 ± 18.0	47.1 ± 11.7	47.6 ± 13.9	50.5 ± 15.9	63.0 ± 20.9^**^	67.7 ± 16.5^***^	**0.0009**
	8. Pleasure of orgasm	42.9 ± 19.4	44.2 ± 13.8	45.6 ± 14.6	42.5 ± 14.9	56.0 ± 17.5^***^	59.5 ± 13.2^***^	** <0.0001**
	9. Overall pleasure of lovemaking	47.8 ± 18.7	46.7 ± 10.9	49.6 ± 14.2	49.5 ± 14.2	56.9 ± 16.7^*^	59.1 ± 15.3^*^	**0.0372**
	10. Partner's overall pleasure of lovemaking	52.2 ± 20.7	50.8 ± 9.2	52.8 ± 14.7	52.5 ± 12.5	58.8 ± 16.6	59.1 ± 17.3	0.1797
	SLQ scale score (mean of item 1-10)	45.0 ± 15.1	44.3 ± 7.5	46.3 ± 11.9	46.8 ± 12.8	56.2 ± 12.5^***^	60.9 ± 12.7^***^	** <0.0001**

*Data are mean ± SD. Product effect was evaluated by ANCOVA with stratification of gender. Post-hoc test with Bonferroni adjustment was applied to variables with significant group difference for further pair-wise group comparison at day 60*.

* *P < 0.05*,

** *P < 0.01, and*

*** *P < 0.001 compared with placebo group. Numbers in bold indicate statistical significance*.

### Blood Biomarkers

The concentrations of blood biomarkers were comparable at baseline (Table 6). After the product intervention, both the low-dose and high-dose groups had significantly lower blood ammonia (*P* < 0.01 and *P* < 0.05, respectively) and blood lactic acid concentration (*P* < 0.05 and *P* < 0.01, respectively) than the placebo group. The intervention of the product led to a trend of increasing testosterone, although the difference did not reach statistical significance (*P* = 0.0743).

**Table 6:** Group differences in blood biomarkers.

	**Baseline**			**Day 60**			**Product effect *p*-value**
	**Placebo (*n* = 58)**	**Low dose (*n* = 58)**	**High dose (*n* = 59)**	**Placebo (*n* = 58)**	**Low dose (*n* = 58)**	**High dose (*n* = 59)**	
							
Blood ammonia (μmol/L)	44.85 ± 16.13	45.14 ± 14.17	45.99 ± 14.26	47.05 ± 13.10	41.53 ± 11.43^**^	41.60 ± 11.75^*^	**0.0110**
Blood glucose (mmol/L)	4.81 ± 0.54	4.74 ± 0.52	4.72 ± 0.60	4.76 ± 0.52	4.81 ± 0.44	4.80 ± 0.51	0.3164
Free fatty acid (μmol/L)	565.3 ± 161.4	556.3 ± 165.6	550.8 ± 153.1	543.1 ± 184.8	525.1 ± 149.4	520.6 ± 159.1	0.8194
Creatine kinase (U/L)	94.62 ± 30.43	91.24 ± 36.97	96.39 ± 36.34	99.48 ± 34.83	94.84 ± 33.99	98.63 ± 31.61	0.4238
C-reactive protein (mg/L)	1.25 ± 1.46	1.31 ± 1.44	1.04 ± 0.93	1.30 ± 1.44	1.43 ± 1.43	1.23 ± 1.43	0.9650
Blood lactic acid (mmol/L)	2.53 ± 0.45	2.50 ± 0.38	2.51 ± 0.38	2.59 ± 0.50	2.40 ± 0.43^*^	2.39 ± 0.33^**^	**0.0059**
Estradiol—female (pg/ml)	32.1	25.6	28.1	38.1	33.2	36.7	0.7620
Testosterone—male (ng/ml)	4.07 ± 1.50	4.34 ± 1.19	4.05 ± 1.16	3.87 ± 1.18	4.51 ± 1.30	4.35 ± 0.95	0.0743

*Data are mean ± SD. Product effect was evaluated by ANCOVA. Post-hoc test with Bonferroni adjustment was applied to variables with significant group difference for further pair-wise group comparison at day 60*.

* *P < 0.05 and*

** *P < 0.01 compared with placebo group*.

*Estradiol concentration data was highly skewed. Median was reported and non-parametric method was used for group comparison. Numbers in bold indicate statistical significance*.

Logistic regression was used to evaluate the effect of the changes in blood biomarkers on the remission and relief of CFS symptoms (overall effectiveness). The results are shown in Table 7. The changes in blood glucose and lactic acid concentrations were significantly associated with the severity of CFS symptoms in both univariate analysis (*P* = 0.0058 and 0.0061, respectively) and multivariate analysis (OR = 3.062 and 0.334, respectively; *P* = 0.0075 and 0.0108, respectively). This indicates that the odds of ameliorating CFS tripled with one unit increase of blood glucose but were threefold less with one unit increase of circulating lactic acid concentration. Pearson correlation analysis and significance tests were further used to evaluate the correlation between the changes in blood biomarkers and the changes in the Chalder fatigue questionnaire scores (Supplementary Table 2). The change in blood lactic acid concentration was significantly correlated with the total physical fatigue score (*R* = 0.1639, *P* = 0.0302), Q1-problems of tiredness (*R* = 0.1886, *P* = 0.0166), and Q2-need to rest more (*R* = 0.2254, *P* = 0.0027). Despite the statistically significant *P*-values, the correlation coefficients showed a low linear relationship (*R* <0.30), suggesting a weak strength of the association. Therefore, caution needs to be imposed in interpreting the data, and the clinical significance of this formula warrants further evaluation in a clinical trial with expanded sample size and extended follow-up duration.

**Table 7:** Univariate and multivariate associations between product effectiveness and the changes in blood biomarkers.

**Covariates**	**Univariate**		**Multivariate**	
	**OR (95% CI)**	***p*-value**	**OR (95% CI)**	***p*-value**
Blood ammonia	1.001 (0.984–1.019)	0.8654	0.999 (0.980–1.018)	0.9272
Blood glucose	3.021 (1.378–6.624)	0.0058	3.062 (1.349–6.951)	**0.0075**
Free fatty acid	1.000 (0.998–1.002)	0.8537	1.000 (0.998–1.002)	0.8503
Creatine kinase	1.002 (0.988–1.017)	0.7376	1.006 (0.991–1.021)	0.4467
C-reactive protein	0.846 (0.610–1.175)	0.3173	0.825 (0.582–1.171)	0.2820
Blood lactic acid	0.313 (0.136–0.717)	0.0061	0.334 (0.144–0.776)	**0.0108**
Estradiol (female)	1.001 (0.994–1.007)	0.8276	N/A	N/A
Testosterone (male)	1.188 (0.801–1.764)	0.3916	N/A	N/A

*Logistic regression was used to evaluate the associations between product effectiveness (remission and relief) and the changes in blood biomarkers after the intervention. OR, odds ratio; CI, confidence interval; N/A, not applicable. Numbers in bold indicate statistical significance*.

### Product Safety Evaluation

ALT, AST, GGT, and BUN were analyzed as liver and kidney function safety biomarkers. All subjects had normal levels of ALT, AST, GGT, and BUN at initial enrollment. Table 8 shows the summary statistics of liver and kidney function biomarkers for the 175 subjects who completed the study and had measurements through the course of intervention. All biomarkers were within normal ranges for all subjects, and no significant difference among study groups was observed among the three groups throughout the study.

**Table 8:** Summary of liver and kidney function biomarkers.

		**Baseline**			***p*-value**
		**Placebo**	**Low dose**	**High dose**	
Baseline	Alanine Aminotransferase-ALT (U/L)	17.52 ± 7.37	17.98 ± 6.48	17.78 ± 6.91	0.9224
	Aspartate Aminotransferase-AST (U/L)	19.93 ± 3.99	19.86 ± 4.35	19.43 ± 3.65	0.8322
	Gamma Glutamyl Transferase-GGT (U/L)	20.97 ± 7.90	20.83 ± 7.76	20.47 ± 7.85	0.8473
	Blood Urea Nitrogen-BUN (mmol/L)	4.88 ± 1.02	4.80 ± 0.95	4.72 ± 0.79	0.5364
Day 30	Alanine Aminotransferase-ALT (U/L)	17.78 ± 6.68	18.27 ± 6.13	18.05 ± 6.10	0.8911
	Aspartate Aminotransferase-AST (U/L)	20.03 ± 4.10	19.93 ± 3.94	19.59 ± 3.26	0.8873
	Gamma Glutamyl Transferase-GGT (U/L)	21.02 ± 7.06	20.66 ± 6.81	20.72 ± 7.26	0.8794
	Blood Urea Nitrogen-BUN (mmol/L)	4.85 ± 0.90	4.82 ± 0.87	4.81 ± 0.81	0.8977
Day 60	Alanine Aminotransferase-ALT (U/L)	18.03 ± 6.03	18.08 ± 5.50	17.95 ± 5.53	0.9842
	Aspartate Aminotransferase-AST (U/L)	20.43 ± 4.91	20.49 ± 9.00	19.38 ± 4.33	0.5032
	Gamma Glutamyl Transferase-GGT (U/L)	21.59 ± 7.22	23.49 ± 9.00	21.21 ± 6.97	0.1880
	Blood Urea Nitrogen-BUN (mmol/L)	4.83 ± 0.85	4.79 ± 0.83	4.77 ± 0.79	0.8599

*Data are mean ± SD. Product effect was evaluated by ANCOVA. Post-hoc test with Bonferroni adjustment was applied to variables with significant group difference for further pair-wise group comparison at day 30 and day 60*.

Among all subjects, seven (3.68%) mild adverse events were reported, including upper respiratory tract infection (*n* = 3), diarrhea (*n* = 1), elevated BUN concentration at Day 30 (*n* = 2), and elevated ALT and AST (*n* = 1). However, none of these adverse events were related to the study products. No severe adverse events were reported in this study.

## Discussion

The etiology of CFS is still unknown; therefore, the diagnosis of CFS is assessed in accordance with symptom-specific criteria (7). The CDC criteria (1994) are the most frequently used case definition for CFS (2). An updated report from the National Academy of Medicine published in 2015 officially classified CFS as a disease, and the diagnostic criteria were updated accordingly (25). However, since this report was released after the initiation and execution of the study, herein the CDC criteria were followed for enrolling CFS subjects. After the intervention of the product, there was a significantly higher effectiveness (remission and relief) rate in the product group compared to the placebo group. Among all eight mentioned symptoms described in the CDC criteria, impaired memory or concentration, muscle pain, unrefreshing sleep, and post-exertional malaise were significantly relieved in the subjects consuming the product. Multiple questionnaires have been previously employed to measure fatigue quantitatively (26), among which the Chalder fatigue questionnaire is the most widely used (20, 21). The 11-item Chalder fatigue questionnaire consists of two sections, one evaluating physical fatigue and the other evaluating mental fatigue, and both sections were included in the current study. These components of the questionnaire were validated by a study involving 361 CFS patients and 1,615 healthy individuals (21). This questionnaire has been shown to have high internal consistency, as indicated by a Cronbach alpha that ranged between 0.86–0.92 (20, 21, 27). One notable limitation of the Chalder fatigue questionnaire is ceiling effects, meaning that patients often record the maximum score on most of the 11 items, leaving no space to indicate a worsening of their fatigue symptoms (28) and making the reliability of the Likert scale questionable. Therefore, we employed a professional physician to validate the severity of CFS to verify the observation of deterioration. By using the Chalder fatigue questionnaire, we observed marginal but statistically significant improvement in the fatigue scores in the product group. However, such improvement may not be clinically meaningful, since according to the PACE trial, an efficacious treatment for CFS should present a difference of 2–4 using a Likert scale (29, 30), but the difference in our current study was <1. Such a small effect size might be due to the fluctuating nature of CFS and the limited time points used to assess CFS in the current study. Therefore, the efficacy of the product against CFS is promising but needs further validation.

QOL is an important outcome evaluated in the CFS research (7). In the currently used WHO-QOL questionnaire, 26 items are divided into four sections, PHYS, PSYCH, SOCIL, and ENVIR (22, 23). This botanical product improved four items (Q3, 10, 16, and 17) in PHYS, two items (Q7 and 26) in PSYCH, one item (Q21) in SOCIL, but no items in ENVIR. Among all the items, Q3 was related to muscle pain; Q7 was related to impaired memory or concentration, and Q16 was related to unrefreshing sleep. These data were consistent with those from the CFS assessment. Poor sleep quality is widely reported in patients with CFS (31), and nocturia has been associated with poor sleep quality (32, 33). In our study, we observed a significantly (*P* < 0.01) improved frequency of nocturia in the subjects of the product group (data not shown). The beneficial effect of this product on the nocturia was mainly attributed to the cistanche, a well-known kidney *yang*-tonifying herb of TCM (12, 34). Improving memory is an original health claim of the product. In the current study, we confirmed the beneficial effects on memory and concentration; these largely depended on the ginkgo (35, 36). Muscle pain was another improved symptom in this study, and this is the first report to show the protective effect of cistanche and ginkgo on muscle pain.

Considering the kidney *yang*-tonifying effect of cistanche, and that the kidney is a critical organ related with sexual function from the TCM perspective (37), we additionally collected an SLQ questionnaire in the current study. A difference between male and female subjects in SLQ questionnaire measurements was observed, i.e., for male subjects all items were improved, whilst female subjects failed on two items, the ease of insertion and partner's overall pleasure during intercourse. This interesting difference suggested that the combination of cistanche and gingko was possibly more effective for men, especially for erectile function. We further examined sex hormones and observed a trend of increase in testosterone after the intervention, but no significant difference was found for estradiol. These data were consistent with previously reported *in vivo* data showing that showed cistanche extract increased testosterone level and improved reproductive dysfunction in rats (38–41). Testosterone is a sex hormone that has been shown to regulate libido, bone mass, muscle mass and strength, and the production of sperm (42). The improved sexual behavior in the male subjects was positively associated with enhanced testosterone, indicating that the tested herbal mixture in the current study may improve the males' sexual capability by moderating the release of testosterone. However, more mechanistic studies are warranted to further validate this speculation. No reports concerning the effects of cistanche on estradiol are available at present.

Lactic acid is produced and accumulated in muscle under the conditions of high energy demand and insufficient oxygen supply (43). The accumulation of lactic acid is commonly observed in CFS subjects (44, 45). Cistanche and gingko extracts were reported to reduce the blood lactic acid levels in mice and rats (11). In the current RCT, the level of blood lactic acid in the high-dose group was 0.2 mmol/L less than that of placebo group (2.39 ± 0.33 vs. 2.59 ± 0.50 mmol/L), an 8% decrease at the end of the intervention. This degree of difference in circulating lactic acid was also observed in subjects involved in sustained handgrip exercise (30% voluntary contraction) for 120 s (rest 1.3 ± 0.29 mmol/L vs. exercise 1.5 ± 0.3 mmol/L) that caused significant fatigue (46). Furthermore, the change in lactic acid concentration was significantly associated with the effectiveness for CFS symptoms in both univariate and multivariate analyses. Pearson correlation and significance tests showed that the change in lactic acid concentration was significantly correlated with the total physical fatigue score as measured by the CFQ. All these data suggested that blood lactic acid could be a potential biomarker to predict the prognosis of CFS.

Ammonia is a waste product of nitrogen-containing compounds such as amino acids and nucleic acids (47). Circulating ammonia accumulation has a significant impact on fatigue due to its capability to disturb neuropsychological function (47) and reduce muscle contraction (48). In the current study, the blood ammonia level in the intervention group (high dose) was 5.5 μmol/L less than in the placebo group. Interestingly, an increase of 10–20 μg/dL (5.8–11.4 μmol/L) blood ammonia was observed in the subjects involved in cycling at 80–100% ventilatory threshold for 15 min, and such an increase led to the onset of fatigue (49).

The reactive oxygen species are known to contribute to fatigue of skeletal muscle through inhibiting calcium sensitivity and depressing force (50). Therefore, the removal of these reactive oxygen species may also alleviate the sensation of fatigue. Ginkgo extract contains flavonol glycosides and terpenoid lactones that are known as antioxidants with the ability to scavenge free radicals and balance the redox status (51). Cistanche contains various bioactive compounds such as phenylethanoid glycosides (e.g., echinacoside), flavonoids, lignin, and alkaloids that render cistanche as a potent antioxidant with the capability to improve mitochondrial function (52). Cistanche was found to enhance ATP generation capacity and mitochondrial electron transport in H9c2 cells (53). Since a functional electron transport chain is essential in preventing overproduction of pyruvate (54) by causing lactic acid accumulation (55), in the current study the decrease of blood lactic acid in the intervention group may be attributed to the cistanche and gingko supplementation.

One limitation of the current study is the deterministic nature of the formula, making it difficult to attribute the observed effects on CFS to any specific constituent; therefore, we cannot estimate the additional improvement that the combination provided in comparison to the individual active compounds (namely, cistanche or ginkgo). However, it is possible that the effects of the combination of cistanche and ginkgo were more potent than those using cistanche or ginkgo alone, since a matrix of compounds may generate synergistic effects (23, 56). Another gap in our knowledge is the specific bioactive compounds contributing to beneficial effects. Considering that cistanche and ginkgo are extracts that mimic whole herbal preparations, herbal extracts would be expected to be more efficacious than single phytochemicals, as exemplified by a report that tomatoes or tomato products showed more potent effects than lycopene in mitigating liver diseases (57, 58) and lung cancer (57, 59, 60). In addition, the assessment of CFS and quality of life was performed by subjective questionnaires instead of objective tests. The current study used the Chalder fatigue questionnaire that was developed to assess the extent and severity of fatigue in both clinical trials and epidemiological studies. The advantage of this questionnaire is that it provides a brief tool for measuring both physical and psychological fatigue with a straightforward answering system. Due to the fluctuating nature of the disease, assessment methods involved in the Chalder fatigue questionnaire may not reflect deterioration in CFS or treatment response when comparing respondents to healthy controls (31), and thus developing a CFS assessment tool with improved reliability is warranted for future studies. The current study employed an experienced physician to validate the severity of CFS based on the diagnostic criteria published by the U.S. CDC (2), and thus we are confident that the data reported in this study were reliable. Memory was assessed as the 11th question (Q11) in the Chalder fatigue questionnaire (Table 3) to help researchers differentiate between fatigue cases and the healthy population. According to Morriss et al. (61), reliability coefficients for Q11 have been high in the studies with CFS patients. Loge et al. (27) also reported high reliability of Q11 in occupational and general population research, with values ranging from 0.90 for the Likert scoring method and 0.83 for the binary scoring method, leading us to believe that the answer to Q11 is a reliable indicator of CFS patients' memory. Moreover, although the group mean differences were statistically significant, they seemed trivial. However, since we used pairwise group comparisons, the criteria for rejecting our hypothesis were stricter compared with Student's *t*-tests.

In conclusion, we carried out a randomized, double-blind, placebo-controlled study using a botanical product containing cistanche and ginkgo extracts and demonstrated that such a product exhibits promising effects in relieving the CFS symptoms and improving the scores of a series of quality of life measurements obtained *via* Chalder fatigue, QOL, and SLQ questionnaires. These findings suggest a botanical-based nutritional supplementation approach in mitigating CFS symptoms and improving quality of life. However, considering the above-mentioned limitations, studies with more comprehensive assessment tools, expanded sample size, and an extended follow-up period are warranted to validate the current findings.

## Data Availability Statement

The raw data supporting the conclusions of this article will be made available by the authors, without undue reservation.

## Ethics Statement

The studies involving human participants were reviewed and approved by IRB of Shanghai Nutrition Society. The patients/participants provided their written informed consent to participate in this study.

## Author Contributions

SLi and JD: designed research. CH, DV, and MM: participated the conception and design of the clinical protocol and sample preparation. JK and JC: analyzed data. LC and SLiu: validated the analysis. JK, JC, and CH: wrote the paper. JD had primary responsibility for final content. All authors read and approved the final manuscript.

## Conflict of Interest

JK, CH, LC, SLiu, DV, MM, and JD are employees of Nutrilite Health Institute, a division of Amway. The remaining authors declare that the research was conducted in the absence of any commercial or financial relationships that could be construed as a potential conflict of interest. The reviewer HG declared a shared affiliation, though no other collaboration, with one of the authors SLi, to the handling Editor.

## Publisher's Note

All claims expressed in this article are solely those of the authors and do not necessarily represent those of their affiliated organizations, or those of the publisher, the editors and the reviewers. Any product that may be evaluated in this article, or claim that may be made by its manufacturer, is not guaranteed or endorsed by the publisher.
