# Supplementary material for: A Botanical Product Containing Cistanche and Ginkgo Extracts Potentially Improves Chronic Fatigue Syndrome Symptoms in Adults: A Randomized, Double-Blind, and Placebo-Controlled Study
Source: Front Nutr. 2021 Nov 26;8:658630. doi: 10.3389/fnut.2021.658630 (PMC8662561; doi:10.3389/fnut.2021.658630)
Supplement: Supplementary file 1 [file Data_Sheet_1.PDF]

Supplementary Table 1. Summary of the change of individual symptoms of chronic fatigue syndrome from baseline to day 60

		Placebo (n=58)	Low dose (n = 58)		High dose (n = 59)		Product effect *p*-value
Impaired memory or concentration	Relief	8 (13.8%)	26 (44.8%)	***	32 (54.2)	***	<.0001
	No change	50 (86.2%)	32 (55.2%)		27 (45.8%)		
Muscle pain	Relief	8 (86.2%)	25 (43.1%)	***	29 (49.2%)	***	0.0001
	No change	50 (86.2%)	33 (56.9%)		30 (50.9%)		
Unrefreshing sleep	Relief	3 (5.2%)	22 (37.9%)	***	27 (45.8%)	***	<.0001
	No change	55 (94.8%)	36 (62.1%)		32 (54.2%)		
Post-exertional malaise	Relief	5 (8.6%)	22 (37.9%)	***	25 (42.4%)	***	<.0001
	No change	53 (91.4%)	36 (62.1G)		34 (57.6G)		
Sore throat	Relief	5 (8.6%)	11 (19.0%)		14 (23.7%)		0.1444
	No change	47 (81.0%)	45 (77.6%)		42 (71.2%)		
	Worsen	6 (10.3%)	2 (3.5%)		3 (5.1%)		
Tender cervical or axillary lymph nodes	Relief	0 (0.0%)	2 (3.5%)		1 (1.7%)		0.5451
	No change	57 (98.3%)	55 (94.8%)		58 (98.3%)		
	Worsen	1 (1.7%)	1 (1.7%)		0 (0.0%)		
Multiple joint pain	Relief	0 (0.0%)	3 (5.2%)		1 (1.7%)		0.2225
	No change	58 (100%)	54 (93.1%)		58 (98.3%)		
	Worsen	0 (0.0%)	1 (1.7%)		0 (0.0%)		
New headaches	Relief	5 (8.6%)	8 (13.8%)		8 (13.6%)		0.6828
	No change	48 (82.8%)	47 (81.0%)		49 (83.1%)		
	Worsen	5 (8.6%)	3 (5.2%)		2 (3.4%)		

Data are frequency (%). Product effect was evaluated by chi-square test. Post-hoc test with Bonferroni adjustment was applied to variables with significant group difference for further pair-wise group comparison. $^{***}P < 0.001$ compared with placebo group.

Supplementary Table 2. Pearson correlation coefficients between changes in blood biomarkers and changes in Chadler fatigue questionnaire scores (day 60 – baseline)

	**Blood ammonia**	**Blood glucose**	**Free fatty acid**	**Creatine kinase**	**C-reactive protein**	**Blood lactic acid**	**Estradiol (female)**	**Testosterone (male)**
**Physical fatigue (total score)**	0.0158	-0.0964	-0.1128	0.0187	0.0304	0.1639	-0.0293	-0.0184
	0.8352	0.2043	0.1371	0.8059	0.6899	0.0302*	0.7825	0.8683
1. Do you have problems with tiredness?	0.1077	-0.0293	-0.0761	-0.0115	0.0020	0.1886	-0.1145	-0.2004
	0.1559	0.7008	0.3170	0.8802	0.9788	0.0166*	0.2799	0.0676
2. Do you need to rest more?	-0.0477	-0.0823	-0.0907	-0.0167	0.0143	0.2254	0.0471	-0.0320
	0.5311	0.2788	0.2327	0.8260	0.8515	0.0027**	0.6576	0.7724
3. Do you feel sleepy or drowsy?	0.0705	-0.0657	0.0572	-0.0332	0.0044	0.0677	-0.1806	0.0255
	0.3539	0.3874	0.4524	0.6630	0.9545	0.3733	0.0867	0.8182
4. Do you have problems starting things?	0.0255	-0.0879	-0.0438	-0.0291	0.0660	0.1418	-0.1114	0.1705
	0.7376	0.2477	0.5653	0.7027	0.3857	0.0612	0.2932	0.1210
5. Do you lack energy?	0.0105	-0.0097	-0.0977	0.0268	0.0088	0.1179	-0.0261	0.0945
	0.8903	0.8991	0.1984	0.7248	0.9080	0.1201	0.8059	0.3926
6. Do you have less strength in your muscles?	-0.0577	-0.1033	-0.1196	0.0616	0.0544	-0.0331	0.1225	-0.1049
	0.4480	0.1736	0.1150	0.4185	0.4745	0.6641	0.2472	0.3423
7. Do you feel weak?	-0.0647	-0.0676	-0.1402	0.0884	0.0106	0.0017	0.1018	-0.1408
	0.3952	0.3740	0.0643	0.2445	0.8888	0.9825	0.3368	0.2014
**Mental fatigue (total score)**	0.0477	-0.1410	-0.0605	-0.0327	-0.0202	0.0430	-0.0991	-0.0939
	0.5307	0.0628	0.4267	0.6674	0.7909	0.5717	0.3500	0.3955
8. Do you have difficulties concentrating?	0.0585	-0.1486	-0.0864	-0.0011	-0.0147	0.1159	-0.0483	-0.1135
	0.4423	0.0496*	0.2554	0.9888	0.8471	0.1267	0.6495	0.3040
9. Do you make slips of the tongue when speaking?	0.1023	-0.0467	0.1295	-0.1501	0.0633	0.0004	-0.1869	-0.0054
	0.1779	0.5393	0.0877	0.0474*	0.4056	0.9962	0.0760	0.9611
10. Do you find it more difficult to find the correct word?	-0.0066	-0.0919	-0.1238	0.0089	0.0149	-0.0585	-0.1504	-0.1822
	0.9314	0.2266	0.1025	0.9071	0.8449	0.4422	0.1548	0.0973
11. How is your memory?	-0.0185	-0.0591	-0.0437	0.0248	-0.0794	0.0193	0.0436	-0.1851
	0.8085	0.4374	0.5655	0.7445	0.2962	0.8002	0.6817	0.0919

$^{**}P < 0.01$; $^{*}P < 0.05$.
